# Supplementary material for: Metabolic crosstalk between skeletal muscle cells and liver through IRF4-FSTL1 in nonalcoholic steatohepatitis
Source: Nat Commun. 2023 Sep 28;14:6047. doi: 10.1038/s41467-023-41832-3 (PMC10539336; doi:10.1038/s41467-023-41832-3)
Supplement: Supplementary file 1 — Supplementary Information [file 41467_2023_41832_MOESM1_ESM.pdf]

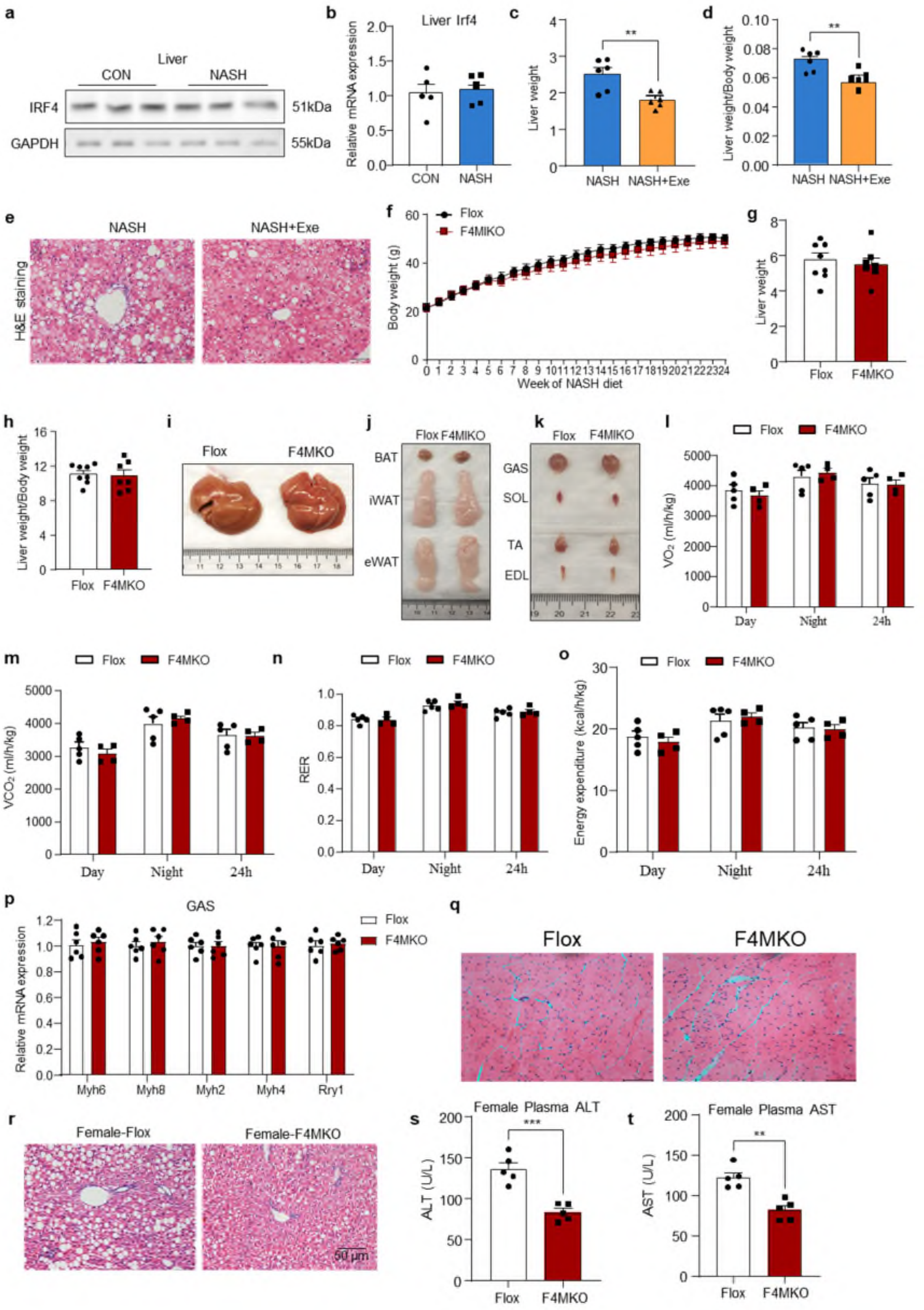

Supplementary Figure 1. IRF4 expression has no alteration in liver of NASH. (**a** and **b**) Western blot (n=3 biologically independent animals) and QPCR (CON, n=5 biologically independent animals; NASH, n=6 biologically independent animals) analysis of the expression of IRF4 in liver of control (CON) and NASH mice. (**c** and **d**) The weight and ratio of liver of NASH and NASH+Exe mice (n=6 biologically independent animals). (**e**) H&E staining of liver in NASH and NASH+Exe mice (n=3 biologically independent animals.

Scale bars, 50 μm). (**f**) The body weight of Flox and F4MKO mice on 24-weeks NASH diet. (**g** and **h**) The weight and ratio of liver of Flox and F4MKO mice on 24-weeks NASH diet (Flox, n=8 biologically independent animals; F4MKO, n=7 biologically independent animals). (**i**-**k**) The images of different liver, fat and skeletal muscle of Flox and F4MKO mice on 24-weeks NASH diet. (**l**-**o**) The O_2 consumption, CO_2 production, respiratory exchange ratio, and energy expenditure of male Flox and F4MKO mice on 24-weeks NASH diet (Flox, n=5 biologically independent animals; F4MKO, n=4 biologically independent animals). (**p**) Relative mRNA expression of marker gene of muscle fiber type in male Flox and F4MKO mice on 24-weeks NASH diet (n=6 biologically independent animals). (**q**) H&E staining of gastrocnemius in male Flox and F4MKO mice on 24-weeks NASH diet (n=3 biologically independent animals. Scale bars, 100 μm). (**r**) H&E staining of liver in female Flox and F4MKO mice (n=3 biologically independent animals. Scale bars, 50 μm). (**s** and **t**) Serum ALT and AST levels in female Flox and F4MKO mice on 24-weeks NASH diet (n=5 biologically independent animals). All results were shown as mean ± SEM, $^{*}p<0.05$, $^{**}p<0.01$, $^{***}p<0.001$. A two-tailed Student t test was used for statistical analysis. Source data are provided as a Source Data file.

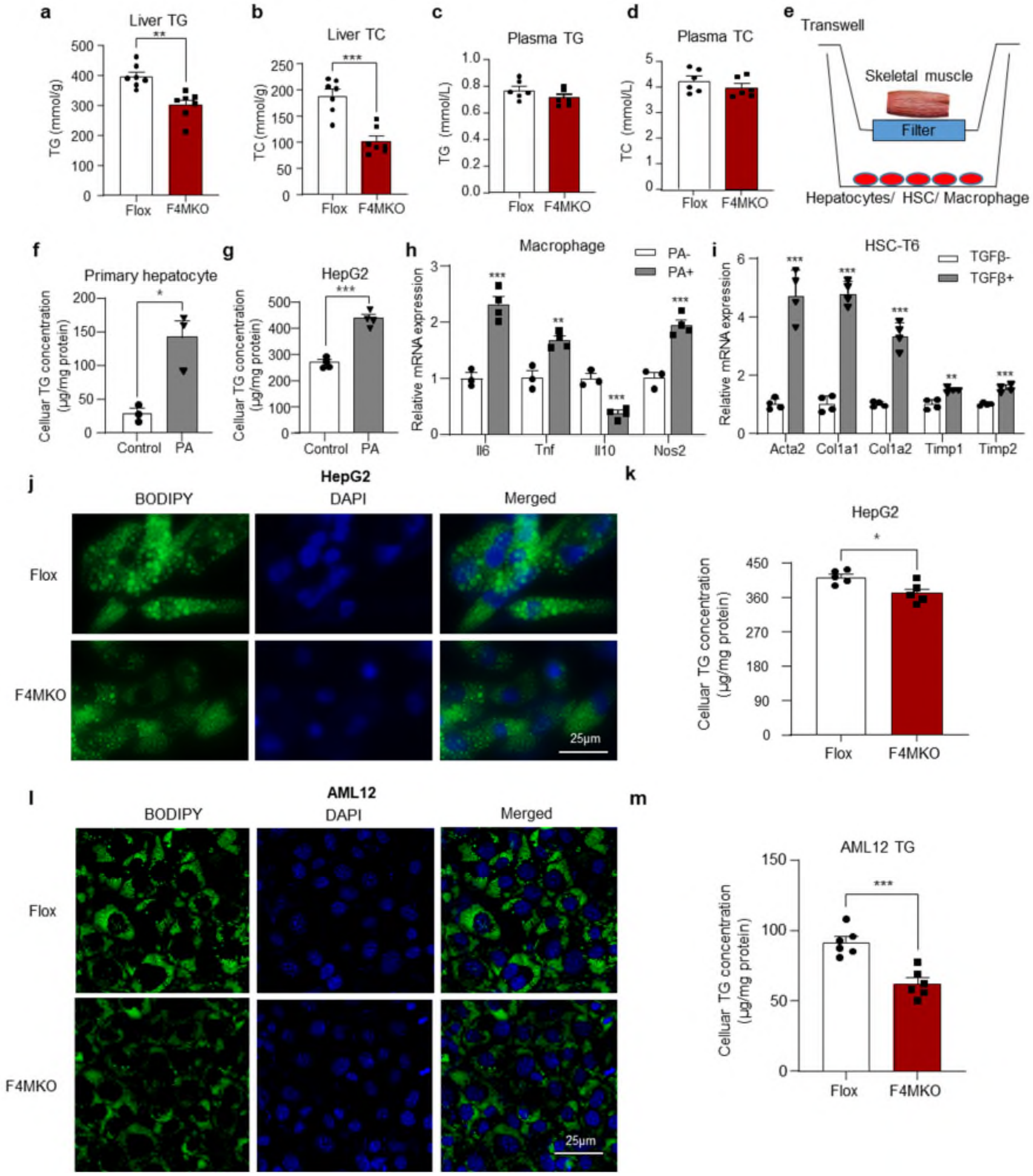

Supplementary Figure 2. Generation of in vitro cell models to mimic NASH in vivo. (**a** and **b**) Liver TG and TC in male Flox and F4MKO mice on NASH diet (n=7 biologically independent animals). (**c** and **d**) Plasma TG and TC in male Flox and F4MKO mice on NASH diet (n=6 biologically independent animals). (**e**) The model of co-culture. (**f**) Cellular TG level of primary hepatocytes (n=3 biologically independent cells). (**g**) TG level of HepG2 cells (n=4 biologically independent cells). (**h**) Relative mRNA expression of inflammatory genes of macrophages treated with 200 μM PA (PA-, n=3 biologically independent cells; PA-, n=4 biologically independent cells). (**i**) Relative mRNA expression of fibrosis genes of HSC-T6 cells treated with 10ng/ml rTGFβ (n=4 biologically independent cells). (**j** and **l**) Immunofluorescence of HepG2 and AML12 cells co-cultured with gastrocnemius from Flox and F4MKO mice (n=3 biologically independent cells). HepG2 and AML12 cells treated with 200 μM PA were co-cultured with gastrocnemius from Flox and F4MKO mice for 24 h.

(k) Cellular TG level of HepG2 cells from j (n=5 biologically independent cells). (**k** and **m**) Cellular TG level of AML12 cells from l (n=6 biologically independent cells). All results were shown as mean ± SEM, **p*<0.05, ***p*<0.01, ****p*<0.001. A two-tailed Student t test was used for statistical analysis. Source data are provided as a Source Data file.

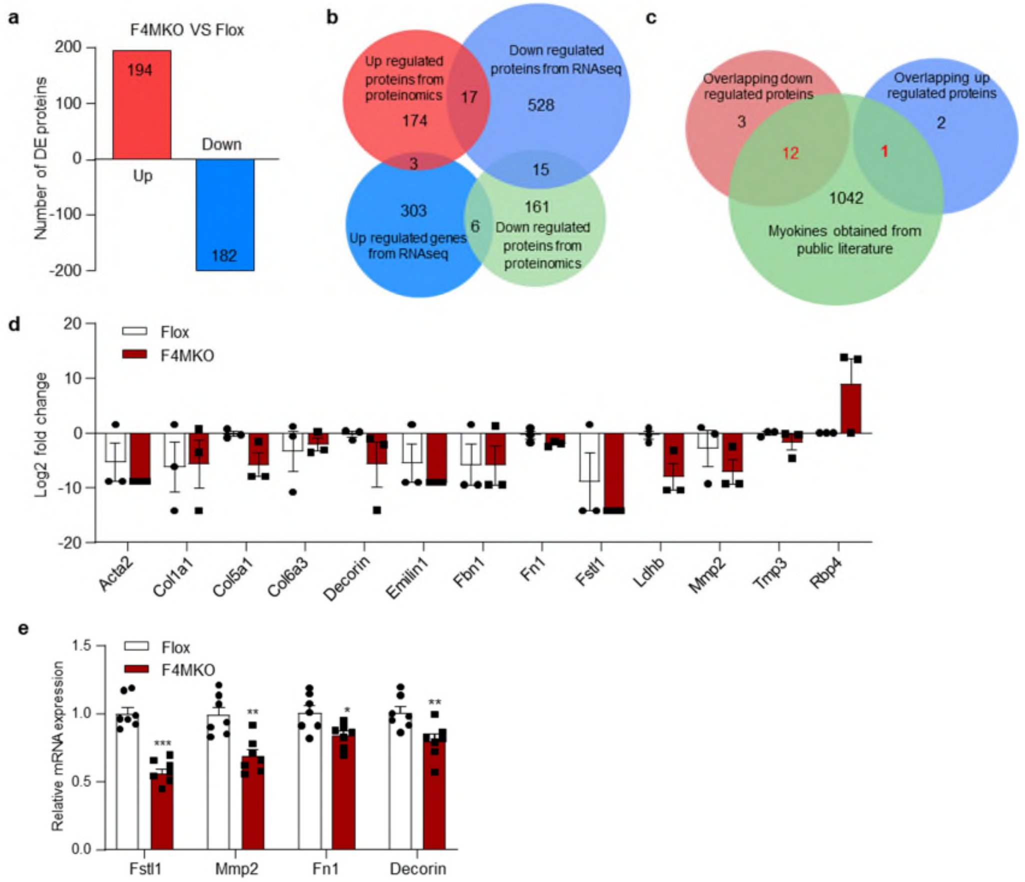

Supplementary Figure 3. Screening myokines responsible for IRF4-mediated NASH progression. (**a**) Proteomics showing differently expressed protein numbers between the serum of Flox and F4MKO mice fed with 24-weeks NASH diet (n=3 biologically independent animals). (**b**) Overlap of differently expressed proteins from proteomics and differently expressed genes from RNAseq data. (upregulated and downregulated proteins from proteinomics were shown in red and green respectively; upregulated and downregulated genes from RNAseq data were shown in bright blue and light blue respectively). (**c**) Overlap of different expressed proteins and myokines obtained from public literatures (upregulated and downregulated proteins from proteinomics were shown in red and blue respectively; myokines obtained from public literatures were shown green). (**d**) Relative level of differently expressed myokines from proteomic data (relative to Flox group). (**e**) Differently expressed genes encoding myokines in the NASH muscle of Flox and F4MKO mice validated by QPCR (n=7 biologically independent animals). All results were shown as mean ± SEM, *p<0.05, **p<0.01, ***p<0.001. A two-tailed Student t test was used for statistical analysis. Source data are provided as a Source Data file.

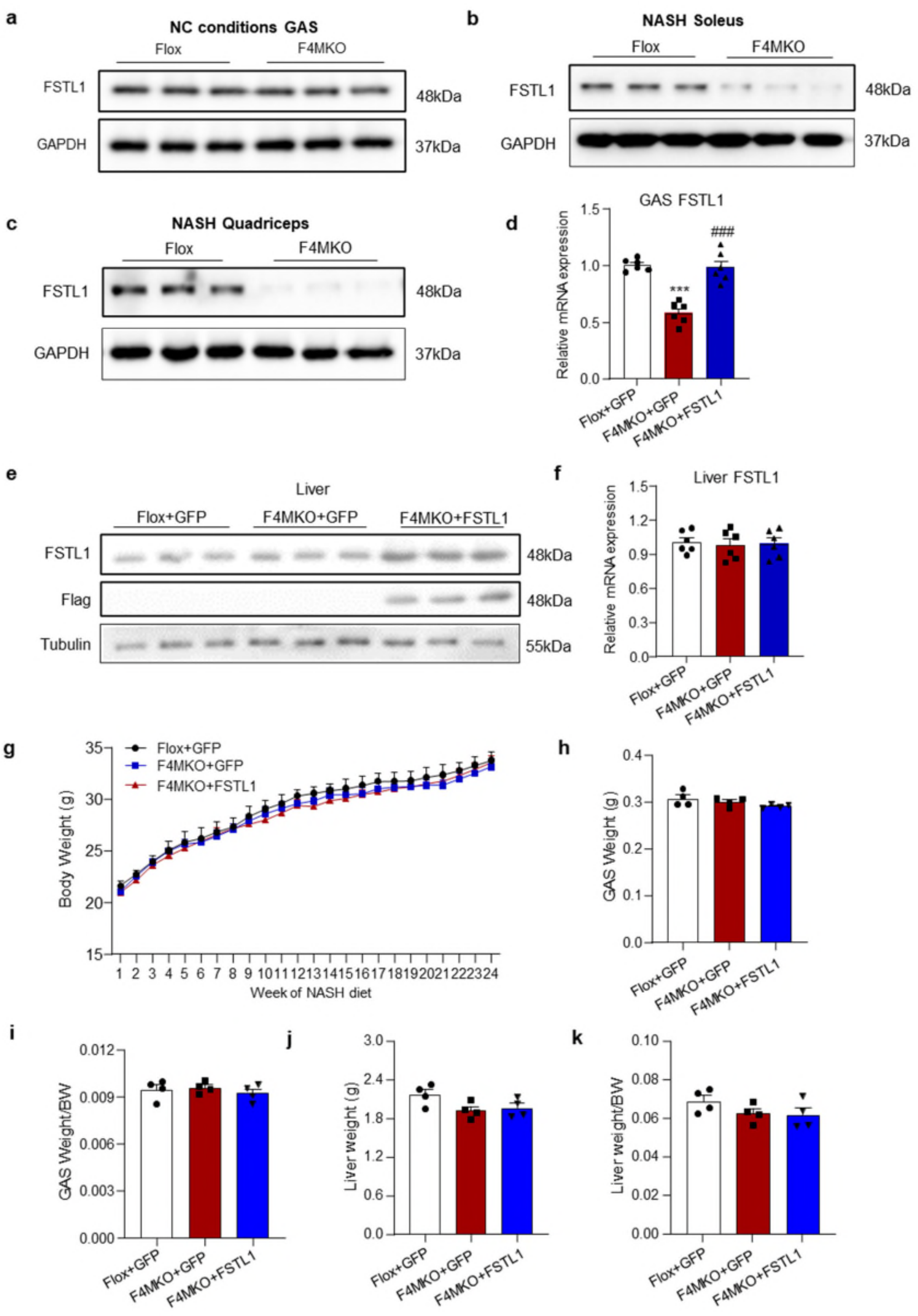

Supplementary Figure 4. FSTL1 derived from skeletal muscle is responsible for IRF4-mediated NASH progression. (**a**) Western blot analysis of the expression of FSTL1 in gastrocnemius from Flox and F4MKO mice under normal chow diet condition (n=3 biologically independent animals). (**b** and **c**) Western blot analysis of the expression of FSTL1 in soleus and quadriceps from Flox and F4MKO mice on 24-weeks NASH diet (n=3 biologically independent animals). (**d**) Relative mRNA expression of FSTL1 in GAS of

Flox+GFP, F4MKO+GFP, and F4MKO+FSTL1 mice on 24-weeks NASH diet (n=6 biologically independent animals). (**e**) Western blot analysis of the expression of FSTL1-FLAG in liver of Flox+GFP, F4MKO+GFP, and F4MKO+FSTL1 mice on 24-weeks NASH diet (n=3 biologically independent animals). (**f**) QPCR analysis of the expression of FSTL1 in the liver of Flox+GFP, F4MKO+GFP, and F4MKO+FSTL1 mice on 24-weeks NASH diet (n=6 biologically independent animals). (**g**) The body weight of Flox+GFP, F4MKO+GFP, and F4MKO+FSTL1 mice on NASH diet (n=4 biologically independent animals). (**h** and **i**) The weight and ratio of liver on NASH diet (n=4 biologically independent animals). (**j** and **k**) The weight and ratio of GAS on NASH diet (n=4 biologically independent animals). All results were shown as means ± SEM. $^{***}p<0.001$, compared with Flox+GFP group; $^{###}p<0.001$, compared with F4MKO+GFP group. The One-way ANOVA followed by Bonferroni post-tests was used for statistical analysis. Source data are provided as a Source Data file.

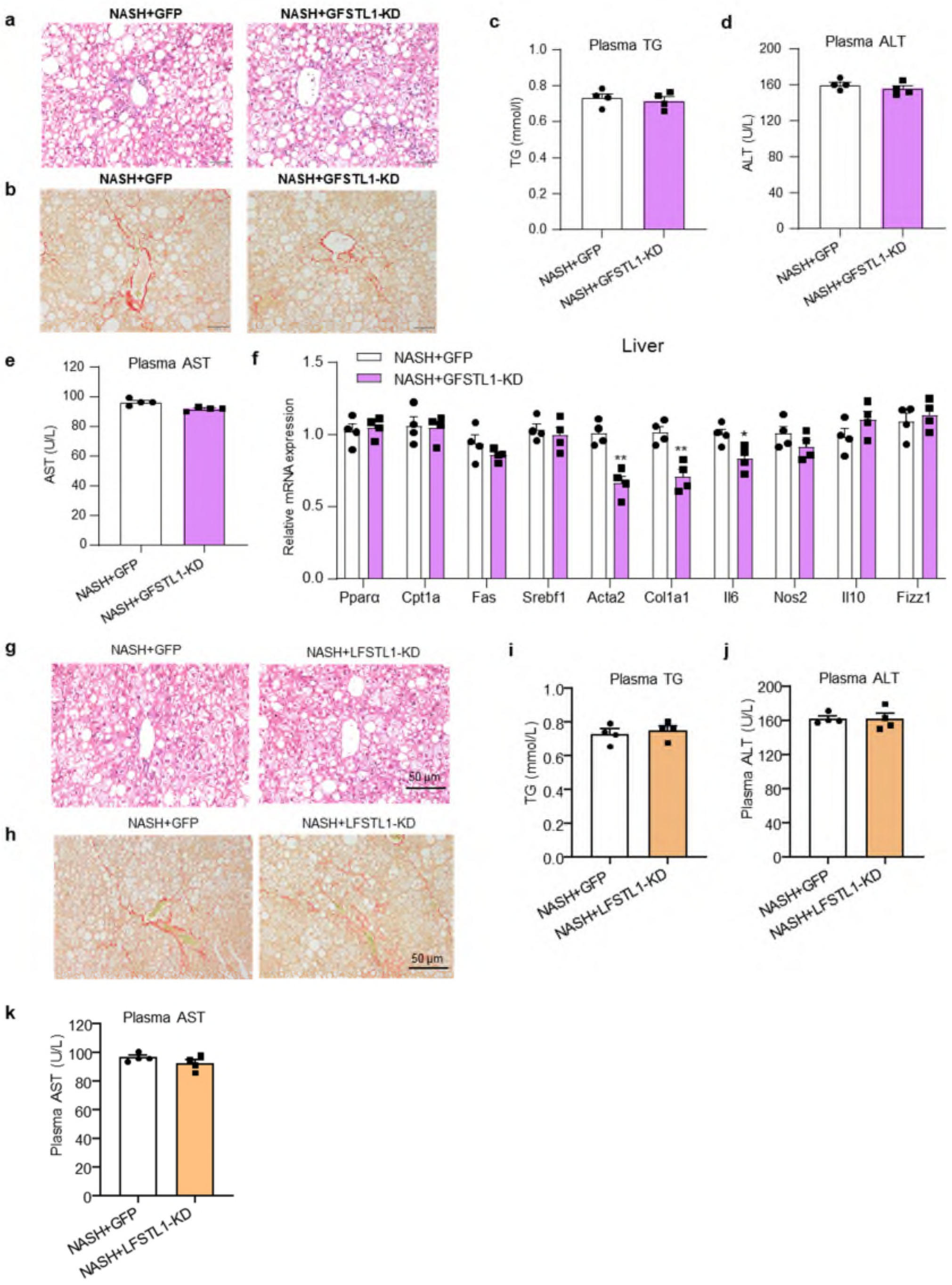

Supplementary Figure 5. Knockdown FSTL1 in GAS, but not liver, alleviated liver fibrosis. (**a** and **b**) H&E and Sirius red staining of liver in NASH+GFP, NASH+GFSTL1-KD (gastrocnemius Fstl1 knock down) mice (n=3 biologically independent animals. Scale bars, 50 μm). (**c**-**e**) Plasma TG, ALT, and AST in NASH+GFP, NASH+GFSTL1-KD mice (n=4 biologically independent animals). (**f**) Relative mRNA expression of lipid metabolism related genes, fibrosis genes, and inflammatory genes in NASH liver from NASH+GFP, NASH+GFSTL1-KD mice (n=4 biologically independent animals). (**g** and **h**) H&E and Sirius Red staining of liver in NASH+GFP, NASH+LFSTL1-KD (liver Fstl1 knock down) mice (n=3

biologically independent animals. Scale bars, 50 μm). (**i-k**) Plasma TG, ALT, and AST in NASH+GFP and NASH+LFSTL1-KD mice (n=4 biologically independent animals). All results were shown as mean ± SEM. A two-tailed Student t test was used for statistical analysis. Source data are provided as a Source Data file.

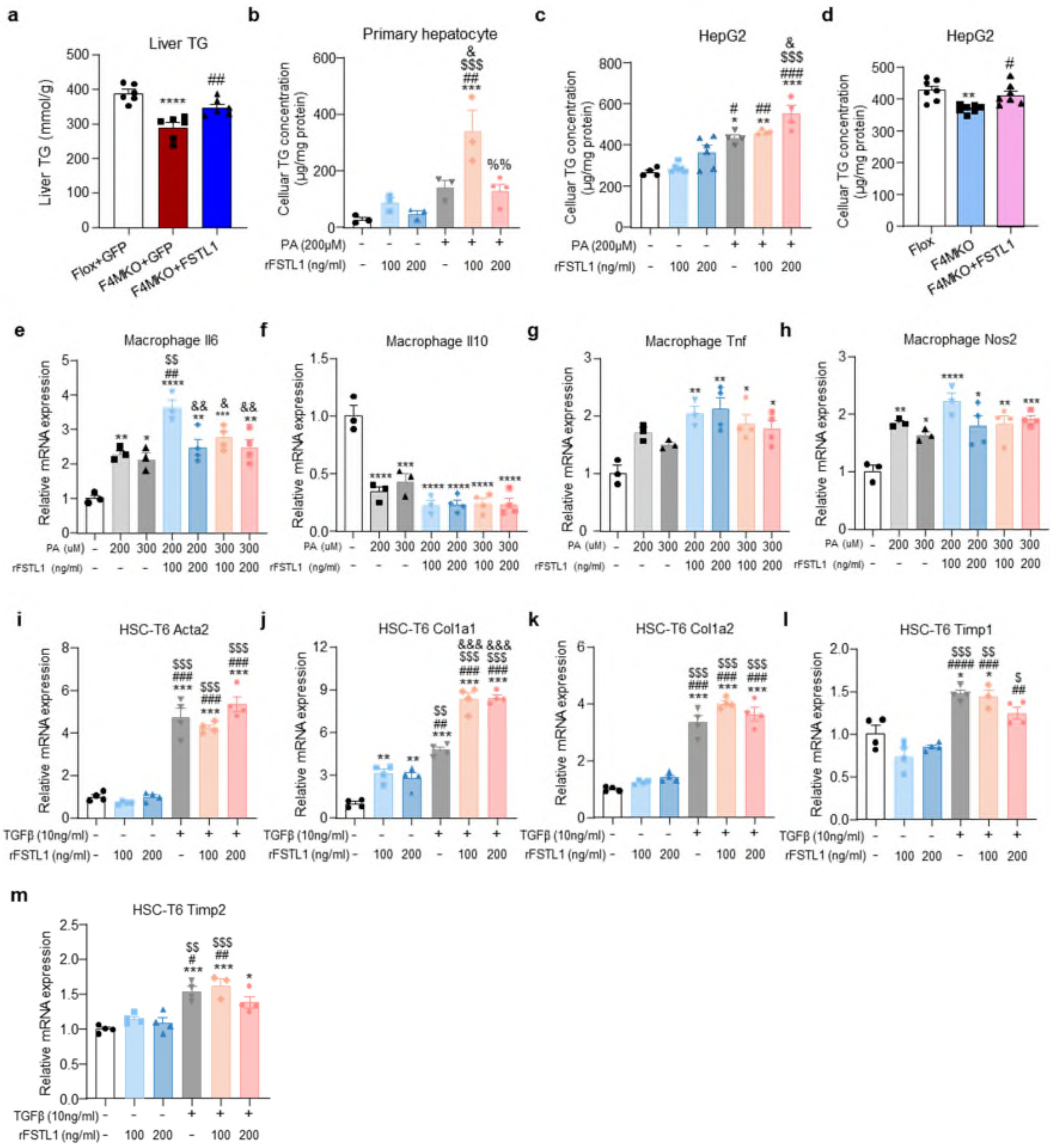

Supplementary Figure 6. Obtaining the optimal working concentration of rFSTL1 on different cell types. (**a**) Liver TG in Flox+GFP, F4MKO+GFP and F4MKO+FSTL1 mice on NASH diet (n=6 biologically independent animals). (**b**) Cellular TG of primary hepatocytes treated with/without rFSTL1. (**c**) Cellular TG HepG2 cells treated with/without rFSTL1. (**d**) Cellular TG level of HepG2 cells co-cultured with GAS from Flox or F4MKO mice (n=7 biologically independent cells). HepG2 cells, treated with 25 mM glucose and 200 μM PA, were co-cultured with GAS from Flox or F4MKO mice for 48 h. Then HepG2 cells were co-cultured with GAS from Flox and F4MKO mice for 48 h and treated with or without 200 ng/ml rFSTL1. (**e**-**h**) Relative mRNA expression of inflammatory genes in macrophages treated with/without rFSTL1. (**i**-**m**) Relative mRNA expression of fibrosis genes in HSC-T6 cells treated with/without rFSTL1. All results were shown as mean ± SEM. $^{***}p$<0.001, compared with the Flox+GFP group; $^{\#}p$<0.05, compared with the F4MKO+GFP (a). $^{*}p$<0.05, $^{**}p$<0.01, $^{***}p$<0.001, compared with the PA$^-$ rFSTL1$^-$ group; $^{\#}p$<0.05, $^{\#\#}p$<0.01, $^{\#\#\#}p$<0.001, compared with the PA$^-$ rFSTL1 100 group; $^{\$\$}p$<0.01, $^{\$\$\$}p$<0.001, compared with the PA$^-$

rFSTL1 200 group; $^{\&}p<0.05$, $^{\&\&\&}p<0.001$, compared with the PA^+ $rFSTL1^-$ group; $^{\%\%}p<0.01$, compared with the PA^+ rFSTL1 100 group (b,c and e-h). $^{**}p<0.01$, compared with the Flox group; $^{\#}p<0.05$, compared with the F4MKO (d). $^{*}p<0.05$, $^{**}p<0.01$, $^{***}p<0.001$, compared with the $TGF\beta^-$ $rFSTL1^-$ group; $^{\#\#}p<0.01$, compared with the $TGF\beta^-$ rFSTL1 100 group; $^{\$\$}p<0.01$, compared with the $TGF\beta^-$ rFSTL1 200 group; $^{\&\&\&}p<0.001$, compared with the TGFβ 10 $rFSTL1^-$ group (i-m). A two-way ANOVA followed by Bonferroni post-tests was used for statistical analysis. Source data are provided as a Source Data file.

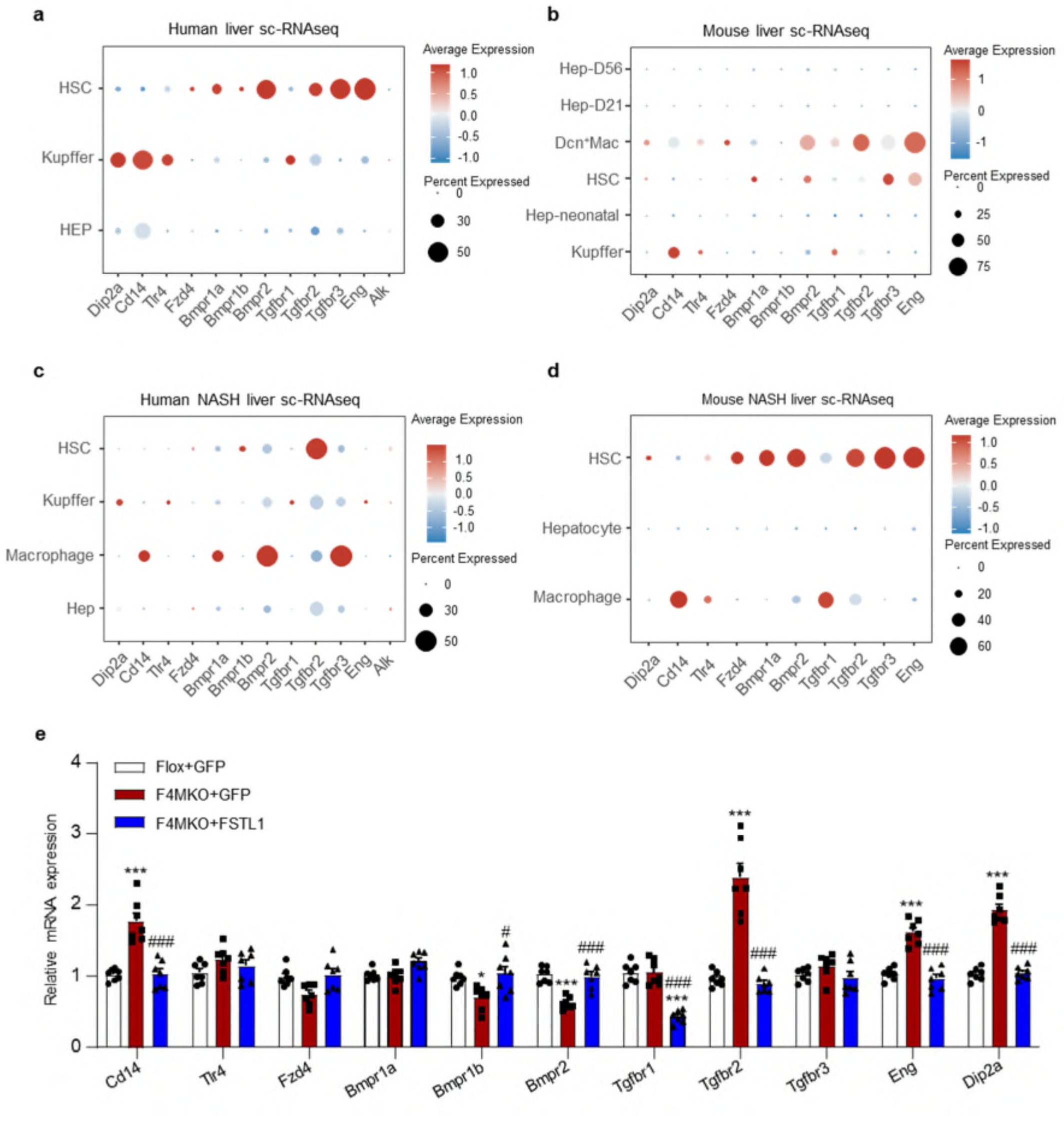

Supplementary Figure 7. Expression of FSTL1 receptors in liver. (**a** and **b**) FSTL1 receptors expression from human and mouse liver sc-RNAseq data. (**c** and **d**) FSTL1 receptors expression from human and mouse NASH liver sc-RNAseq data. (**e**) Relative mRNA expression of FSTL1 receptors in Flox+GFP, F4MKO+GFP and F4MKO+FSTL1 mice on NASH diet (n=7 biologically independent animals). All results were shown as mean ± SEM. $^{*}p<0.05$, $^{***}p<0.001$, compared with the Flox+GFP group; $^{\#}p<0.05$, $^{\#\#\#}p<0.001$, compared with the F4MKO+GFP, a two-way ANOVA followed by Bonferroni post-tests was used for statistical analysis.

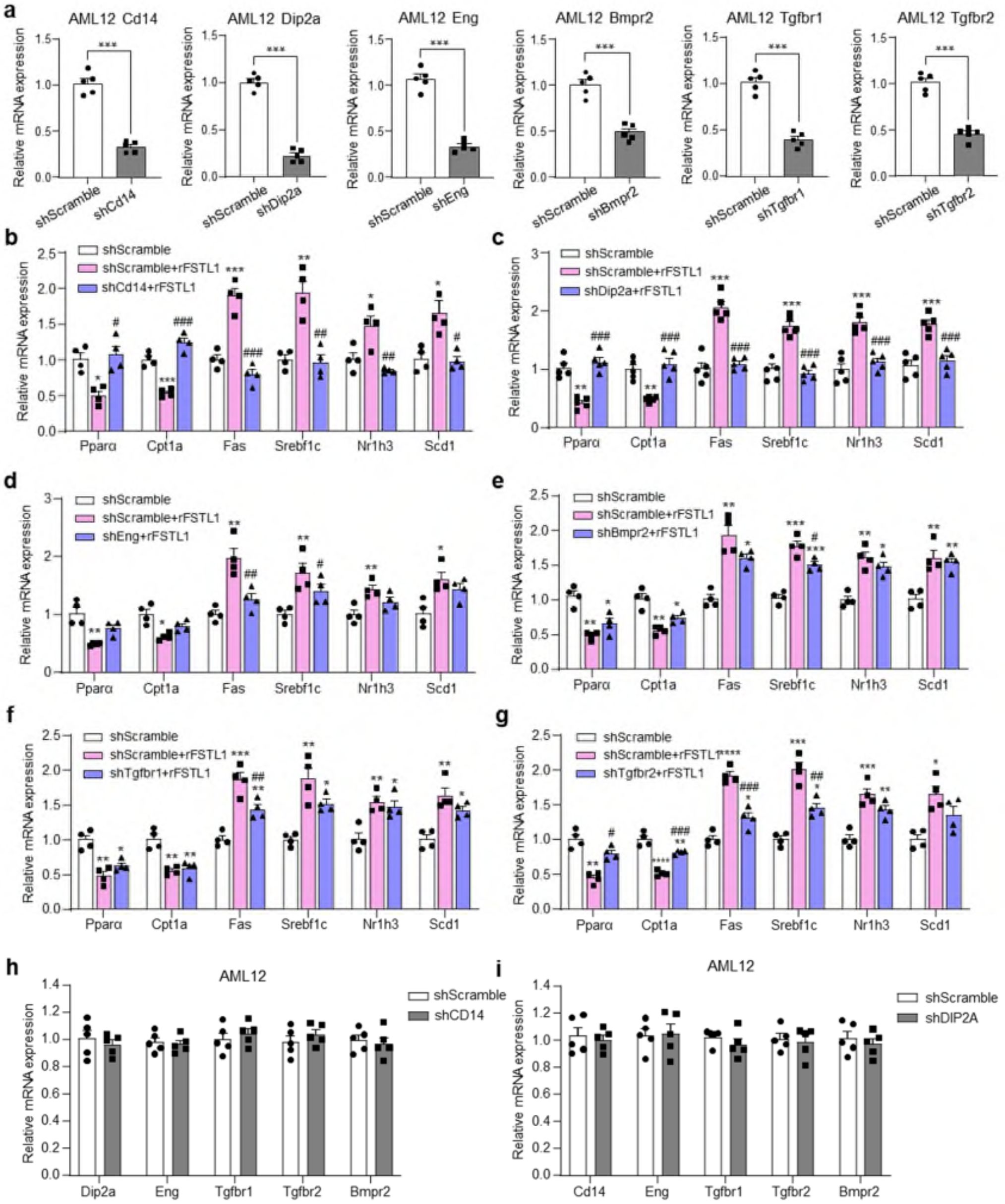

Supplementary Figure 8. FSTL1 exerts its function on hepatocytes through DIP2A and CD14. (**a**) The efficiency of FSTL1 receptors knockdown (n=5 biologically independent cells). (**b**-**g**) Relative mRNA expression of lipid metabolism related genes in indicated groups (n=4 biologically independent cells). AML12 cells were infected with lentivirus of FSTL1 receptors and then treated with 200 μM PA for 24h. In shScramble+rFSTL1 and shReceptors+rFSTL1 groups, AML12 cells were additionally treated with 100 ng/ml rFSTL1. (**h** and **i**) Relative mRNA expression of other FSTL1 receptors in scramble and shDIP2A or shCD14 group (n=5 biologically independent cells). All results were shown as mean ± SEM. $^{*}p$<0.05, $^{**}p$<0.01, $^{***}p$<0.001, compared with the shScramble group, $^{\#}p$<0.05, $^{\#\#}p$<0.01, $^{\#\#\#}p$<0.001, compared with the shScramble+rFSTL1 group. A two-way ANOVA followed by Bonferroni post-tests was used for statistical analysis. Source data are provided as a Source Data file.

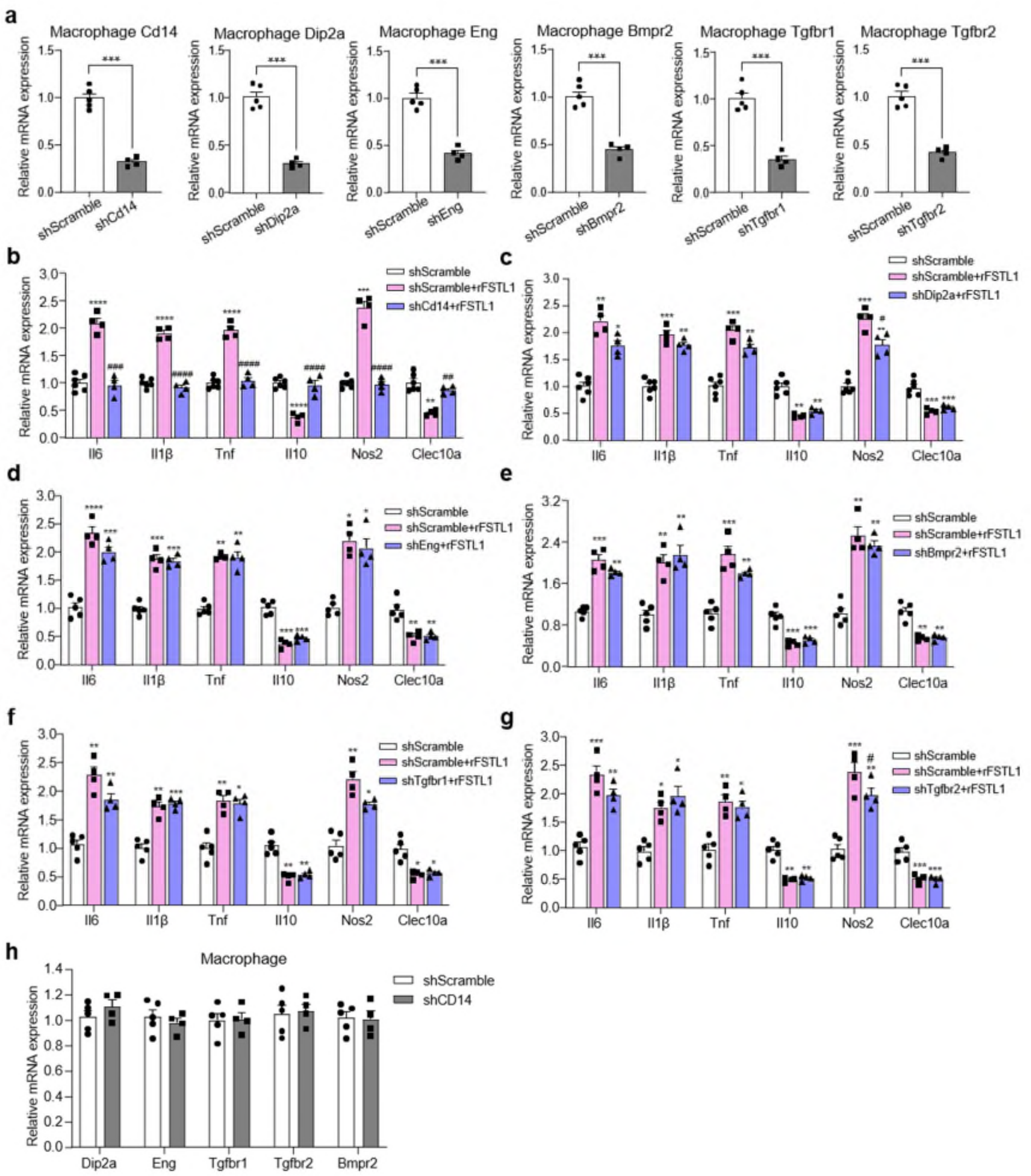

Supplementary Figure 9. FSTL1 exerts its function on macrophages through CD14. (**a**) The efficiency of FSTL1 receptors knockdown (shScramble, n=5 biologically independent cells; n=4 biologically independent cells for others). (**b-g**) Relative mRNA expression of inflammatory genes in indicated groups. Macrophages were infected with lentivirus of FSTL1 receptors and then treated with 200 μM PA for 24h. In shScramble+rFSTL1 and shReceptors+rFSTL1 groups, macrophages were additionally treated with 100 ng/ml rFSTL1. (**h**) Relative mRNA expression of other FSTL1 receptors in scramble and shCD14 group (shScramble, n=5 biologically independent cells; shCD14, n=4 biologically independent cells). All results were shown as mean ± SEM. $^{*}p$<0.05, $^{**}p$<0.01, $^{***}p$<0.001 , compared with the shScramble group, $^{\#}p$<0.05, $^{\#\#}p$<0.01, $^{\#\#\#}p$<0.001, compared with the shScramble+rFSTL1 group. A two-way ANOVA followed by Bonferroni post-tests was used for statistical analysis. Source data are provided as a Source Data file.

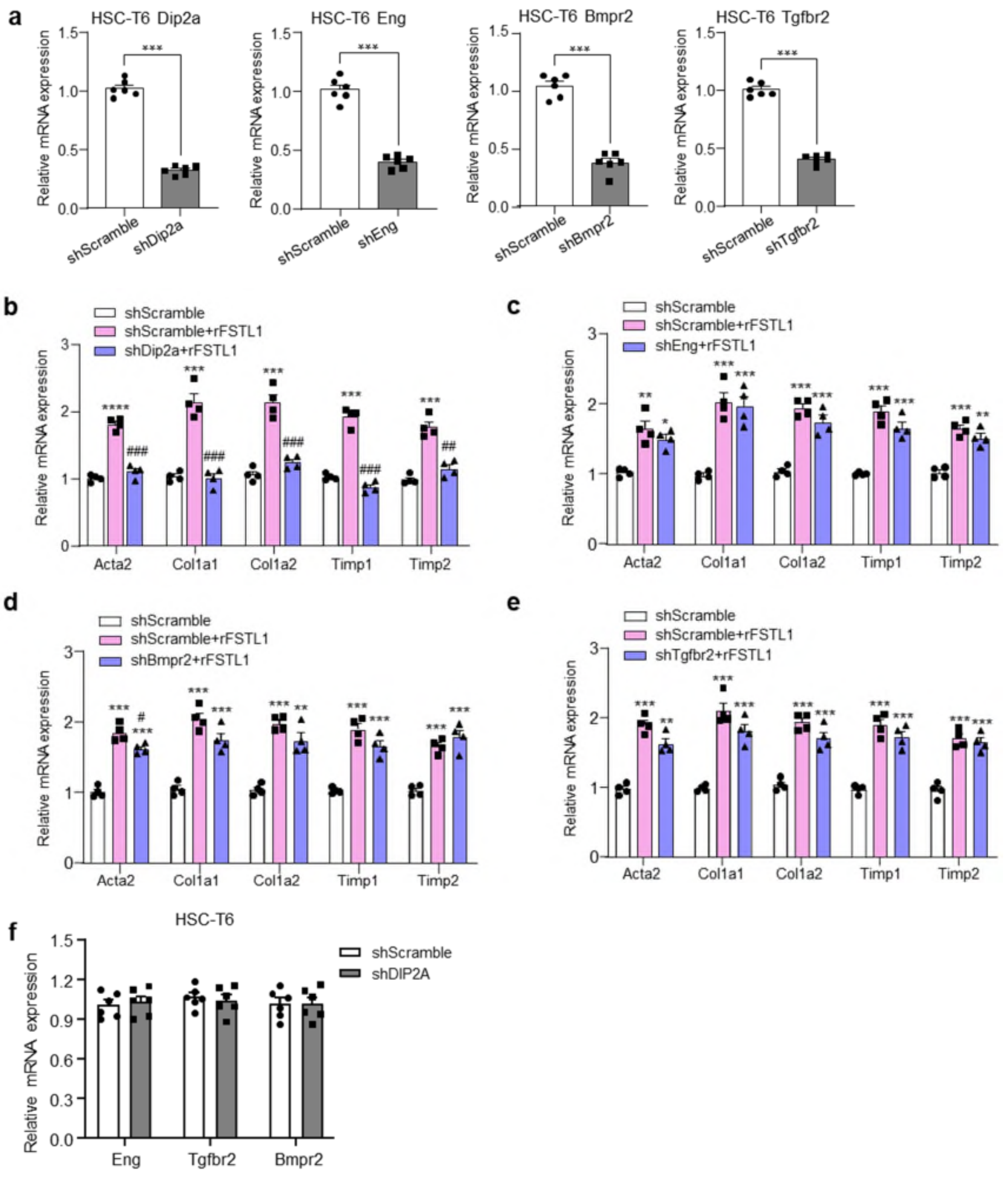

Supplementary Figure 10. FSTL1 exerts its function on HSCs through DIP2A. (**a**) The efficiency of FSTL1 receptors knockdown (n=6 biologically independent cells). (**b**-**e**) Relative mRNA expression of inflammatory genes in indicated groups (n=4 biologically independent cells). HSC-T6 cells were infected with lentivirus of FSTL1 receptors and then treated with 10ng/ml TGFβ for 24h. In shScramble+rFSTL1 and shReceptors+rFSTL1 groups, HSC-T6 cells were additionally treated with 100 ng/ml rFSTL1. (**f**) Relative mRNA expression of other FSTL1 receptors in scramble and shDIP2A group (n=6 biologically independent cells). All results were shown as mean ± SEM. $^{**}p<0.01$, $^{***}p<0.001$, compared with the shScramble group, $^{\#}p<0.05$, $^{\#\#}p<0.01$, $^{\#\#\#}p<0.001$, compared with the shScramble+rFSTL1 group. A two-way ANOVA followed by Bonferroni post-tests was used for statistical analysis. Source data are provided as a Source Data file.

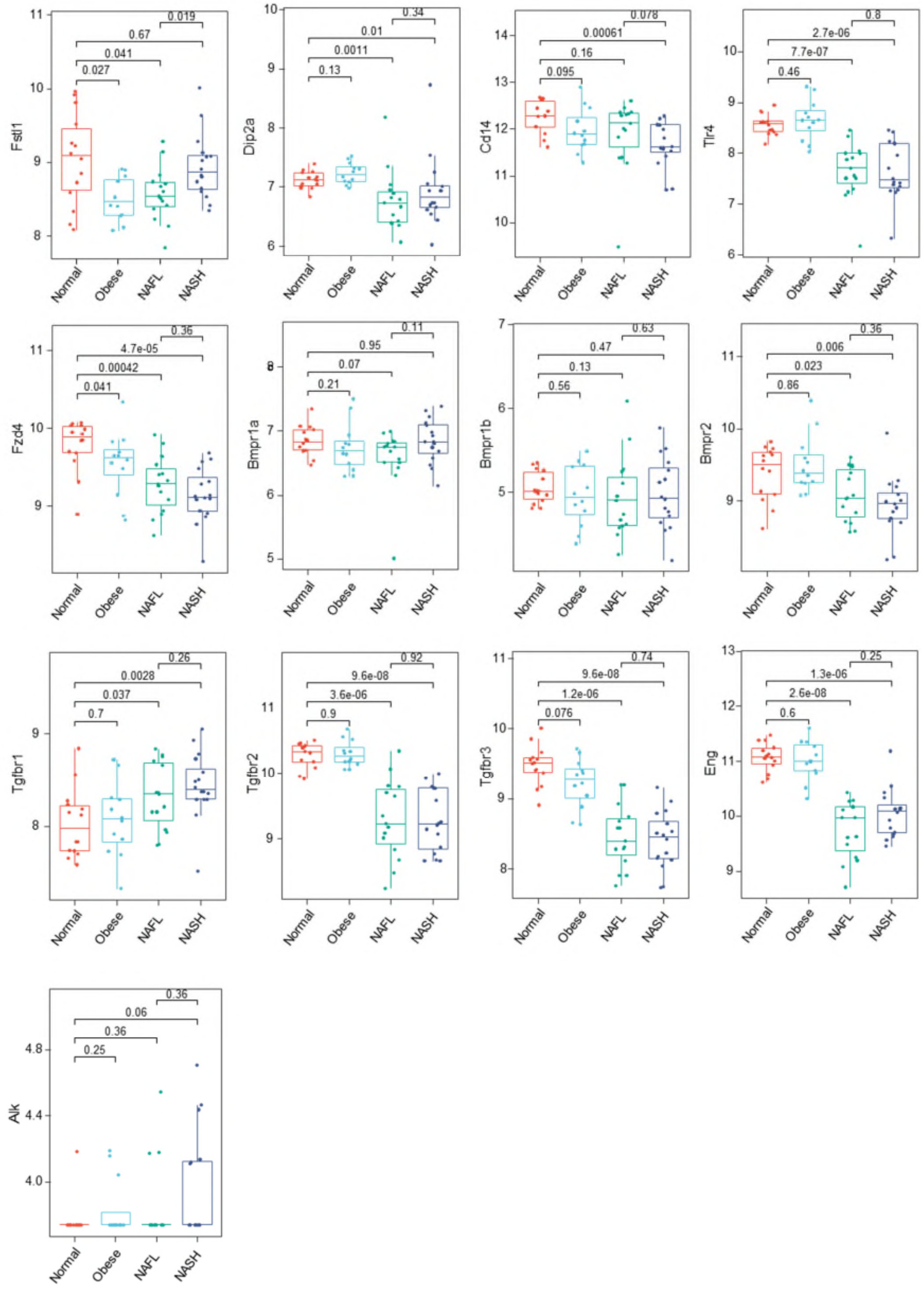

Supplementary Figure 11. Expression of FSTL1 receptors in human liver with indicated disease. All results were shown as mean ± SEM. The Wilcoxon rank sum and signed rank test was used for statistical analysis. Source data are provided as a Source Data file.

Supplementary Table 1 The QPCR primer sequence list

Name	Sequence	Supplier
qPCR mouse *Irf4* forward	CAGGACTACAATCGTGAGGAGG	GENEWIZ
qPCR mouse *Irf4* reverse	GCACATCGTAATCTTGTCTTCCA	GENEWIZ
qPCR mouse *Il6* forward	ATCCAGTTGCCTTCTTGGGACTGA	GENEWIZ
qPCR mouse *Il6* reverse	TAAGCCTCCGACTTGTGAAGTGGT	GENEWIZ
qPCR mouse *Il1β* forward	GAAATGCCACCTTTTGACAGTG	GENEWIZ
qPCR mouse *Il1β* reverse	TGGATGCTCTCATCAGGACAG	GENEWIZ
qPCR mouse *Il10* forward	GCTCTTACTGACTGGCATGAG	GENEWIZ
qPCR mouse *Il10* reverse	CGCAGCTCTAGGAGCATGTG	GENEWIZ
qPCR mouse *Tnf* forward	CAGCGCTGAGGTCAATCTGCC	GENEWIZ
qPCR mouse *Tnf* reverse	TGCCCGGACTCCGCAA	GENEWIZ
qPCR mouse *inos* forward	GTTCTCAGCCCAACAATACAAGA	GENEWIZ
qPCR mouse *inos* reverse	GTGGACGGGTCGATGTCAC	GENEWIZ
qPCR mouse *Clec10a* forward	TGAGAAAGGCTTTAAGAACTGGG	GENEWIZ
qPCR mouse *Clec10a* reverse	GACCACCTGTAGTGATGTGGG	GENEWIZ
qPCR mouse *Ppara* forward	AGAGCCCCATCTGTCCTCTC	GENEWIZ
qPCR mouse *Ppara* reverse	ACTGGTAGTCTGCAAAACCAAA	GENEWIZ
qPCR mouse *Cpt1a* forward	TGCACTACGGAGTCCTGCAA	GENEWIZ
qPCR mouse *Cpt1a* reverse	GGACAACCTCCATGGCTCAG	GENEWIZ
qPCR mouse *Fas* forward	CTGACTCGGCTACTGACACG	GENEWIZ
qPCR mouse *Fas* reverse	TGAGCTGGGTTAGGGTAGGA	GENEWIZ
qPCR mouse *Srebf1* forward	CGGAAGCTGTCGGGGTAG	GENEWIZ
qPCR mouse *Srebf1* reverse	GTTGTTGATGAGCTGGAGCA	GENEWIZ
qPCR mouse *Scd1* forward	CCTCCTGCAAGCTCTACACC	GENEWIZ
qPCR mouse *Scd1* reverse	CAGCCGAGCCTTGTAAGTTC	GENEWIZ
qPCR mouse *Nr1h3* forward	ATCGCCTTGCTGAAGACCTCTG	GENEWIZ
qPCR mouse *Nr1h3* reverse	CTGCTTTGGCAAAGTCTTCCCG	GENEWIZ
qPCR mouse *Acta2* forward	TGCTGACAGAGGCACCACTGAA	GENEWIZ
qPCR mouse *Acta2* reverse	CAGTTGTACGTCCAGAGGCATAG	GENEWIZ
qPCR mouse *Col1a1* forward	GTGCTCCTGGTATTGCTGGT	GENEWIZ
qPCR mouse *Col1a1* reverse	GGCTCCTCGTTTTCCTTCTT	GENEWIZ
qPCR mouse *Col1a2* forward	GTAACTTCGTGCCTAGCAACA	GENEWIZ
qPCR mouse *Col1a2* reverse	CCTTTGTCAGAATACTGAGCAGC	GENEWIZ
qPCR mouse *Timp1* forward	CGAGACCACCTTATACCAGCG	GENEWIZ
qPCR mouse *Timp1* reverse	ATGACTGGGGTGTAGGCGTA	GENEWIZ
qPCR mouse *Timp2* forward	CTGGACGTTGGAGGAAAGAAG	GENEWIZ
qPCR mouse *Timp2* reverse	CTGGGTGATGCTAAGCGTGTC	GENEWIZ
qPCR mouse *Tgfb1* forward	TTGCTTCAGCTCCACAGAGA	GENEWIZ
qPCR mouse *Tgfb1* reverse	TGGTTGTAGAGGGCAAGGAC	GENEWIZ
qPCR mouse *Cd14* forward	CTCTGTCCTTAAAGCGGCTTAC	GENEWIZ
qPCR mouse *Cd14* reverse	GTTGCGGAGGTTCAAGATGTT	GENEWIZ
qPCR mouse *Decorin* forward	TCTTGGGCTGGACCATTTGAA	GENEWIZ
qPCR mouse *Decorin* reverse	CATCGGTAGGGGCACATAGA	GENEWIZ

qPCR mouse *Fibronectin1* forward	ATGTGGACCCCTCCTGATAGT	GENEWIZ
qPCR mouse *Fibronectin1* reverse	GCCCAGTGATTTCAGCAAAGG	GENEWIZ
qPCR mouse *Mmp2* forward	CAAGTTCCCCGGCGATGTC	GENEWIZ
qPCR mouse *Mmp2* reverse	TTCTGGTCAAGGTCACCTGTC	GENEWIZ
qPCR mouse *Tlr4* forward	ATGGCATGGCTTACACCACC	GENEWIZ
qPCR mouse *Tlr4* reverse	GAGGCCAATTTTGTCTCCACA	GENEWIZ
qPCR mouse *Fzd4* forward	AACCTCGGCTACAACGTGAC	GENEWIZ
qPCR mouse *Fzd4* reverse	GGCACATAAACCGAACAAAGGAA	GENEWIZ
qPCR mouse *Bmpr1a* forward	AACAGCGATGAATGTCTTCGAG	GENEWIZ
qPCR mouse *Bmpr1a* reverse	GTCTGGAGGCTGGATTATGGG	GENEWIZ
qPCR mouse *Bmpr1b* forward	CCCTCGGCCCAAGATCCTA	GENEWIZ
qPCR mouse *Bmpr1b* reverse	CAACAGGCATTCCAGAGTCATC	GENEWIZ
qPCR mouse *Bmpr2* forward	TTGGGATAGGTGAGAGTCGAAT	GENEWIZ
qPCR mouse *Bmpr2* reverse	TGTTTCACAAGATTGATGTCCCC	GENEWIZ
qPCR mouse *Tgfbr1* forward	TCTGCATTGCACTTATGCTGA	GENEWIZ
qPCR mouse *Tgfbr1* reverse	AAAGGGCGATCTAGTGATGGA	GENEWIZ
qPCR mouse *Tgfbr2* forward	CCGCTGCATATCGTCCTGTG	GENEWIZ
qPCR mouse *Tgfbr2* reverse	AGTGGATGGATGGTCCTATTACA	GENEWIZ
qPCR mouse *Tgfbr3* forward	GGTGTGAACTGTCACCGATCA	GENEWIZ
qPCR mouse *Tgfbr3* reverse	GTTTAGGATGTGAACCTCCCTTG	GENEWIZ
qPCR mouse *Eng* forward	CCCTCTGCCCATTACCCTG	GENEWIZ
qPCR mouse *Eng* reverse	GTAAACGTCACCTCACCCCTT	GENEWIZ
qPCR mouse *Fstl1* forward	CACGGCGAGGAGGAACCTA	GENEWIZ
qPCR mouse *Fstl1* reverse	TCTTGCCATTACTGCCACACA	GENEWIZ
qPCR mouse *Dip2a* forward	GTGCTCGCGTACTTGGACTT	GENEWIZ
qPCR mouse *Dip2a* reverse	GTGCCCAGAGTAGACGCTG	GENEWIZ
qPCR mouse *Tbp* forward	ACCCTTCACCAATGACTCCTATG	GENEWIZ
qPCR mouse *Tbp* reverse	TGACTGCAGCAAATCGCTTGG	GENEWIZ
qPCR rat *Acta2* forward	TTCCTTCGTGACTACTGCTGAG	GENEWIZ
qPCR rat *Acta2* reverse	CAATGAAAGATGGCTGGAAGAG	GENEWIZ
qPCR rat *Col1a1* forward	CATAAAGGGTCATCGTGGCTTC	GENEWIZ
qPCR rat *Col1a1* reverse	GTGATAGGTGATGTTCTGGGAG	GENEWIZ
qPCR rat *Col1a2* forward	GGTCCAAGAGGAGAACGTGG	GENEWIZ
qPCR rat *Col1a2* reverse	GGGACCTCGGCTTCCAATAG	GENEWIZ
qPCR rat *Timp1* forward	TAAAGCCTGTAGCTGTGCCC	GENEWIZ
qPCR rat *Timp1* reverse	AGCGTCGAATCCTTTGAGCA	GENEWIZ
qPCR rat *Timp2* forward	CCTCTTCGCCCCTTGACAAA	GENEWIZ
qPCR rat *Timp2* reverse	ACGCCCCTCCTCACCTG	GENEWIZ
qPCR rat *B2m* forward	CTACAGCACACGCAGTCTGA	GENEWIZ
qPCR rat *B2m* reverse	GGGACTAAACCTCCAGCCAC	GENEWIZ
qPCR human *Dip2a* forward	CGAAGAGACGTTCTGTCCTTG	GENEWIZ
qPCR human *Dip2a* reverse	GCCGTAAAGAGCCCTCATCT	GENEWIZ
qPCR human *Cd14* forward	ACGCCAGAACCTTGTGAGC	GENEWIZ
qPCR human *Cd14* reverse	GCATGGATCTCCACCTCTACTG	GENEWIZ

qPCR human *Tlr4* forward	TTTGGACAGTTTCCCACATTGA	GENEWIZ
qPCR human *Tlr4* reverse	AAGCATTCCCACCTTTGTTGG	GENEWIZ
qPCR human *Fzd4* forward	GTCTTTCAGTCAAGAGACGCTG	GENEWIZ
qPCR human *Fzd4* reverse	GTTGTGGTCGTTCTGTGGTG	GENEWIZ
qPCR human *Bmpr1a* forward	AGATGACCAGGGAGAAACCAC	GENEWIZ
qPCR human *Bmpr1a* reverse	CAACATTCTATTGTCCGGCGTA	GENEWIZ
qPCR human *Bmpr1b* forward	CTTTTGCGAAGTGCAGGAAAAT	GENEWIZ
qPCR human *Bmpr1b* reverse	TGTTGACTGAGTCTTCTGGACAA	GENEWIZ
qPCR human *Bmpr2* forward	GACAGGAGACCGTAAACAAGG	GENEWIZ
qPCR human *Bmpr2* reverse	CCATATCGACCTCGGCCAATC	GENEWIZ
qPCR human *Tgfbr1* forward	GCTGTATTGCAGACTTAGGACTG	GENEWIZ
qPCR human *Tgfbr1* reverse	TTTTTGTTCCCACTCTGTGGTT	GENEWIZ
qPCR human *Tgfbr2* forward	AAGATGACCGCTCTGACATCA	GENEWIZ
qPCR human *Tgfbr2* reverse	CTTATAGACCTCAGCAAAGCGAC	GENEWIZ
qPCR human *Tgfbr3* forward	GTGTTCCCTCCAAAGTGCAAC	GENEWIZ
qPCR human *Tgfbr3* reverse	AGCTCGATGATGTGTACTTCCT	GENEWIZ
qPCR human *Eng* forward	TGCACTTGGCCTACAATTCCA	GENEWIZ
qPCR human *Eng* reverse	AGCTGCCCACTCAAGGATCT	GENEWIZ
qPCR human *Alk* forward	GCCCTCTGGAAGGTACATTGC	GENEWIZ
qPCR human *Alk* reverse	GAGCACTGTCCAACCATGCTT	GENEWIZ
qPCR human *Fstl1* forward	CCCAGTTGTTTGCTATCAGTCC	GENEWIZ
qPCR human *Fstl1* reverse	TGTAGTTGCTGCCTTTAGAGAAC	GENEWIZ
qPCR human *Tbp* forward	CCACTCACAGACTCTCACAAC	GENEWIZ
qPCR human *Tbp* reverse	CTGCGGTACAATCCCAGAACT	GENEWIZ

Supplementary Table 2 The shRNA sequence list

Name	Squence
Mouse-F-shDip2a	CCGGGGATGGAGACATTTATGAATCCTCGAGGATTCATAAATGTCTCCATCCTTTTTG
Mouse-R-shDip2a	AATTCAAAAAGGATGGAGACATTTATGAATCCTCGAGGATTCATAAATGTCTCCATCC
Mouse-F-shCd14	CCGGATCCCACTCGGAGAAGTTTAACTCGAGTTAAACTTCTCCGAGTGGGATTTTTTG
Mouse-R-shCd14	AATTCAAAAA ATCCCACTCGGAGAAGTTTAACTCGAGTTAAACTTCTCCGAGTGGGAT
Mouse-F-shEng	CCGGCCGTAATGATGGAACTGAGTTCTCGAGAACTCAGTTCCATCATTACGGTTTTTG
Mouse-R-shEng	AATTCAAAAACCGTAATGATGGAACTGAGTTCTCGAGAACTCAGTTCCATCATTACGG
Mouse-F-shBmpr2	CCGGGCCAAGATGAATACAATCAATCTCGAGATTGATTGTATTCATCTTGGCTTTTTG
Mouse-R-shBmpr2	AATTCAAAAAGCCAAGATGAATACAATCAATCTCGAGATTGATTGTATTCATCTTGGC
Mouse-F-shTgfbr1	CCGGCACTTATGCTGATGGTCTATACTCGAGTATAGACCATCAGCATAAGTGTTTTTG
Mouse-R-shTgfbr1	AATTCAAAAACACTTATGCTGATGGTCTATACTCGAGTATAGACCATCAGCATAAGTG
Mouse-F-shTgfbr2	CCGGGAAGGACATCTTCTCCGATATCTCGAGATATCGGAGAAGATGTCCTTCTTTTTG
Mouse-R-shTgfbr2	AATTCAAAAAGAAGGACATCTTCTCCGATATCTCGAGATATCGGAGAAGATGTCCTTC
Rat-F-shDip2a	CCGGGGATGCATGTCATCACCATCCCTCGAGGGATGGTGATGACATGCATCCTTTTTG
Rat-R-shDip2a	AATTCAAAAAGGATGCATGTCATCACCATCCCTCGAGGGATGGTGATGACATGCATCC
Rat-F-shEng	CCGGGCACACCACTACCATCCTTTACTCGAGTAAAGGATGGTAGTGGTGTGCTTTTTG
Rat-R-shEng	AATTCAAAAAGCACACCACTACCATCCTTTACTCGAGTAAAGGATGGTAGTGGTGTGC
Rat-F-shBmpr2	CCGGGGACAATATTATGCTCCAAAGCTCGAGCTTTGGAGCATAATATTGTCCTTTTTG
Rat-R-shBmpr2	AATTCAAAAAGGACAATATTATGCTCCAAAGCTCGAGCTTTGGAGCATAATATTGTCC
Rat-F-shTgfbr2	CCGGGCCCAAGATGCCCATTGTTCACTCGAGTGAACAATGGGCATCTTGGGCTTTTTG
Rat-R-shTgfbr2	AATTCAAAAAGCCCAAGATGCCCATTGTTCACTCGAGTGAACAATGGGCATCTTGGGC
