# Supplementary material for: Metabolic crosstalk between skeletal muscle cells and liver through IRF4-FSTL1 in nonalcoholic steatohepatitis
Source: Nat Commun. 2023 Sep 28;14:6047. doi: 10.1038/s41467-023-41832-3 (PMC10539336; doi:10.1038/s41467-023-41832-3)
Supplement: Supplementary file 3 — Reporting Summary [file 41467_2023_41832_MOESM3_ESM.pdf]

nature portfolio

Corresponding author(s): Xingxing Kong

Last updated by author(s): Sep 06,2023

Reporting Summary

Nature Portfolio wishes to improve the reproducibility of the work that we publish. This form provides structure for consistency and transparency in reporting. For further information on Nature Portfolio policies, see our Editorial Policies and the Editorial Policy Checklist.

Statistics

For all statistical analyses, confirm that the following items are present in the figure legend, table legend, main text, or Methods section.

n/a	Confirmed	
☐	☒	The exact sample size (*n*) for each experimental group/condition, given as a discrete number and unit of measurement
☐	☒	A statement on whether measurements were taken from distinct samples or whether the same sample was measured repeatedly
☐	☒	The statistical test(s) used AND whether they are one- or two-sided<br>*Only common tests should be described solely by name; describe more complex techniques in the Methods section.*
☐	☒	A description of all covariates tested
☐	☒	A description of any assumptions or corrections, such as tests of normality and adjustment for multiple comparisons
☐	☒	A full description of the statistical parameters including central tendency (e.g. means) or other basic estimates (e.g. regression coefficient) AND variation (e.g. standard deviation) or associated estimates of uncertainty (e.g. confidence intervals)
☐	☒	For null hypothesis testing, the test statistic (e.g. *F*, *t*, *r*) with confidence intervals, effect sizes, degrees of freedom and *P* value noted<br>*Give P values as exact values whenever suitable.*
☒	☐	For Bayesian analysis, information on the choice of priors and Markov chain Monte Carlo settings
☒	☐	For hierarchical and complex designs, identification of the appropriate level for tests and full reporting of outcomes
☐	☒	Estimates of effect sizes (e.g. Cohen's *d*, Pearson's *r*), indicating how they were calculated

Our web collection on statistics for biologists contains articles on many of the points above.

Software and code

Policy information about availability of computer code

Data collection	ChemiDoc Imaging System (BIO-RAD) used for collecting images of chemiluminescence blots. ABI 7500 software used for collecting qPCR data.
Data analysis	Graphpad Prism 9.0 used for graphing data and statistical analysis. SPSS software 20.0 used for statistical analysis of human data.

For manuscripts utilizing custom algorithms or software that are central to the research but not yet described in published literature, software must be made available to editors and reviewers. We strongly encourage code deposition in a community repository (e.g. GitHub). See the Nature Portfolio guidelines for submitting code & software for further information.

Data

Policy information about availability of data

All manuscripts must include a data availability statement. This statement should provide the following information, where applicable:

- Accession codes, unique identifiers, or web links for publicly available datasets
- A description of any restrictions on data availability
- For clinical datasets or third party data, please ensure that the statement adheres to our policy

All data supporting the findings of this study are available in the Source Data file. The accession number for the RNA-Seq data reported in this paper is GEO: GSE 216378. All bioinformatics software used in the study are publicly available. Source data are provided with this paper.

Research involving human participants, their data, or biological material

Policy information about studies with human participants or human data. See also policy information about sex, gender (identity/presentation), and sexual orientation and race, ethnicity and racism.

Reporting on sex and gender	A total of 180 subjects were enrolled in this study including 86 males and 94 females.
Reporting on race, ethnicity, or other socially relevant groupings	All subjects were from Zhongshan Hospital, Fudan University. There is no potential bias for recruiting patient cohort.
Population characteristics	Clinical characteristics of subjects (see table below)

Clinical characteristics of subjects

	Control	NAFL	eNASH	fNASH
	N=43	N=43	N=41	N=53
Age (year)	53±15	47±14	40±13	50±13
Sex (male/female)	14/29	26/17	24/17	22/31
BMI (kg/m2)	23.4±2.8	27.2±3.6	29.0±4.9	28.9±3.8
NAS	0	3.1±0.5	5.5±0.6	6.0±0.9
SAF	0	3.6±0.8	6.4±0.7	8.6±1.0

Recruitment	All subjects were from Zhongshan Hospital, Fudan University. A liver biopsy was performed according to the EASL–EASD–EASO clinical practice guidelines to evaluate the severity of liver histology. NAFLD was histologically diagnosed by the presence of ? 5% hepatic steatosis; NAFL was histologically diagnosed by the presence of steatosis without ballooning or lobular inflammation; NASH was histologically diagnosed by the joint presence of steatosis, ballooning, and lobular inflammation. The competing etiologies (chronic viral hepatitis, hypothyroidism, excessive alcohol consumption, drugs leading to steatosis) of steatosis were ruled out based on laboratory examination and liver histology. Subjects with 1) age < 17 years old; 2) type 1 diabetes mellitus, gestational diabetes, and other specific types of diabetes; 3) acute complications of diabetes; 4) severe renal disease or abnormal renal function (Cr?115 μmol/L); or 5) history of malignant tumor, severe mental illness, or parenteral nutrition were excluded.
Ethics oversight	All protocols performed in this study were approved by the ethics committee of the Zhongshan Hospital, Fudan University, and each subject provided written informed consent (NO.B2019-283R).

Note that full information on the approval of the study protocol must also be provided in the manuscript.

Field-specific reporting

Please select the one below that is the best fit for your research. If you are not sure, read the appropriate sections before making your selection.

☒ Life sciences ☐ Behavioural & social sciences ☐ Ecological, evolutionary & environmental sciences

For a reference copy of the document with all sections, see nature.com/documents/nr-reporting-summary-flat.pdf

Life sciences study design

All studies must disclose on these points even when the disclosure is negative.

Sample size	The sample size for each experiment was determined based on our previous experience and experiment. For animal study, to minimize use of animals, only male mice were used for experiments in this study. We used similar sample sizes for different animal groups. For human data, 43 control, 43 NAFL, 41 eNASH, and 53 fNASH subjects were used. Please refer to "Mice" and "Human subjects" in Methods and Materials.
Data exclusions	We excluded animals which developed abnormal disease or other diseases. The exclusion was made before group randomization, experimental intervention and data collection.
Replication	All in vitro experiments were repeated three times independently. Both biological and technical replications were performed.
Randomization	Animals used in experiments of this study were randomly grouped.
Blinding	Immunofluorescent stainings and histological stainings were performed and analyzed in a double-blinded way.

Reporting for specific materials, systems and methods

We require information from authors about some types of materials, experimental systems and methods used in many studies. Here, indicate whether each material, system or method listed is relevant to your study. If you are not sure if a list item applies to your research, read the appropriate section before selecting a response.

Materials & experimental systems

n/a	Involved in the study
☐	☒ Antibodies
☐	☒ Eukaryotic cell lines
☒	☐ Palaeontology and archaeology
☐	☒ Animals and other organisms
☒	☐ Clinical data
☒	☐ Dual use research of concern
☒	☐ Plants

Methods

n/a	Involved in the study
☒	☐ ChIP-seq
☒	☐ Flow cytometry
☒	☐ MRI-based neuroimaging

Antibodies

Antibodies used

anti-IRF4 (Proteintech, 11247-2-AP, dilution 1:1000), anti-FSTL1 (Abcam, ab71548, dilution 1:1000), anti-β-Tublin (Abclonal, AC008, dilution 1:5000), anti-GAPDH (Abway, AB0037, dilution 1:5000), anti-flag (Abclonal, AE005, dilution 1:2000)

Validation

All antibodies were commercially validated for the application used. In addition, all antibodies used for western blotting showed bands at the expected sizes.
anti-IRF4 antibody (https://www.ptgcn.com/products/IRF4-Antibody-11247-2-AP.htm)
anti-FSTL1 antibody (https://www.abcam.cn/products/primary-antibodies/fstl1frp-antibody-ab71548.html)
anti-β-Tublin antibody (https://abclonal.com.cn/catalog/AC008)
anti-GAPDH antibody (http://www.abways.com/showproduct.asp?cid=AB0037)
anti-flag antibody (https://abclonal.com.cn/catalog/AE005)

Eukaryotic cell lines

Policy information about cell lines and Sex and Gender in Research

Cell line source(s)

AML12, Raw264.7, HepG2, HEK 293T from ATCC, HSC-T6 from IMMOCELL.

Authentication

The cell lines were authenticated using STR method by the suppliers.

Mycoplasma contamination

Mycoplasma testing was negative.

Commonly misidentified lines
(See ICLAC register)

No commonly misidentified cell lines were used in the study.

Animals and other research organisms

Policy information about studies involving animals; ARRIVE guidelines recommended for reporting animal research, and Sex and Gender in Research

Laboratory animals

8-week-old male C57BL/6J mice were used in this study. Myl-Cre mice and IRF4 flox/flox mice were all bred on a C57BL/6J background. Mice were maintained under a 12-hours light/12-hours dark cycle at constant temperature (23°C) and humidity (50–60%) with free access to food and water.

Wild animals

Wild animals were not involved.

Reporting on sex

Experiments were conducted in both male and female mice, and the sex is indicated in the relevant sections (e.g figures, legends etc)

Field-collected samples

Field-collected samples were not involved.

Ethics oversight

All research protocols in this study were approved by the Institutional Animal Care and Use Committee of Shanghai university of sport.

Note that full information on the approval of the study protocol must also be provided in the manuscript.
